# Supplementary figures and images for: The Role of Receptor Tyrosine Kinases in Lassa Virus Cell Entry
Source: Viruses. 2020 Aug 6;12(8):857. doi: 10.3390/v12080857 (PMC7472032; doi:10.3390/v12080857)

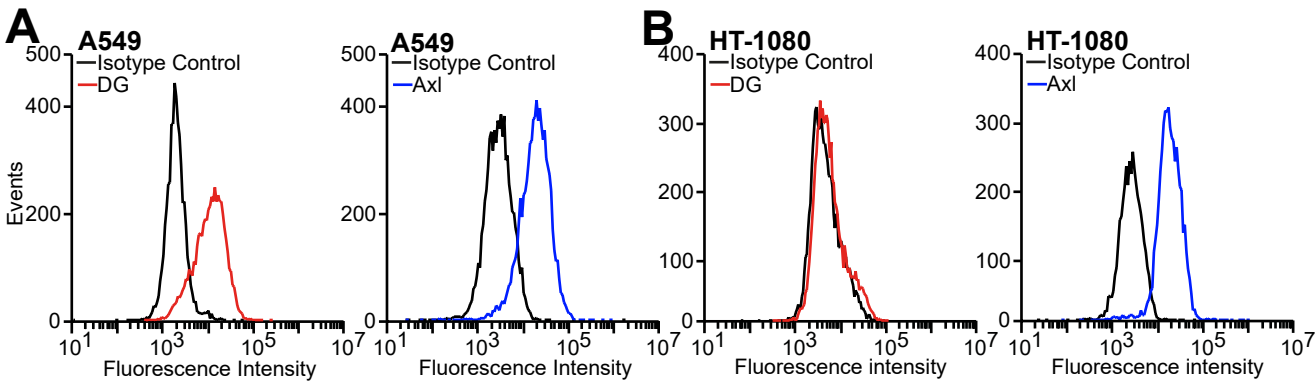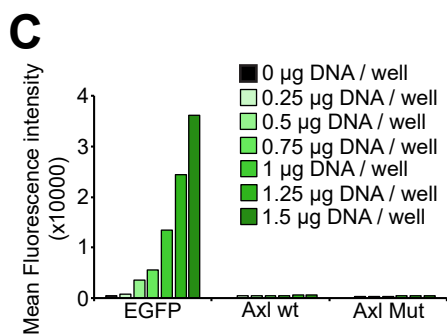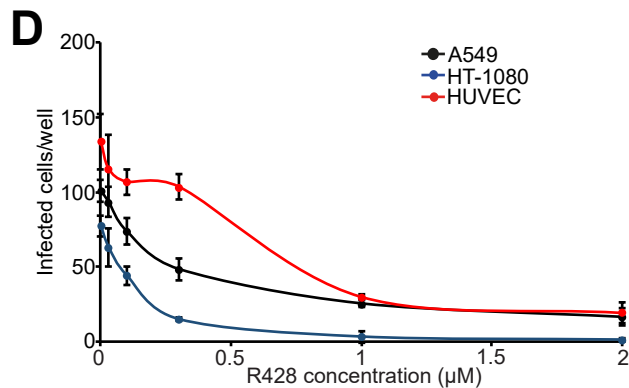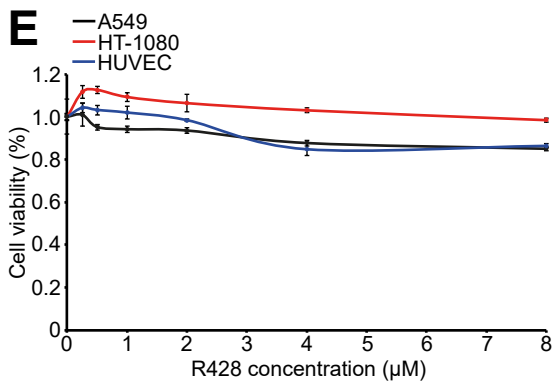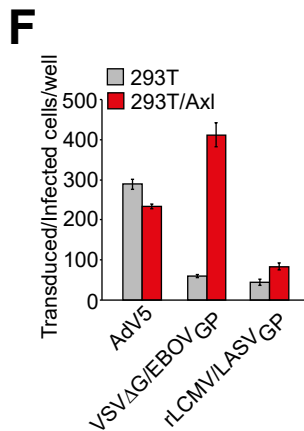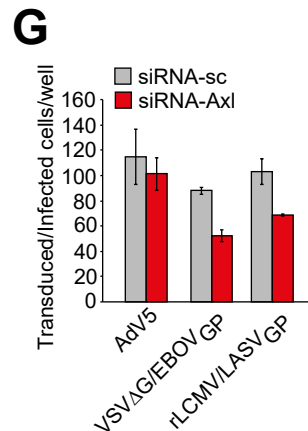

Supplement: Supplementary file 1 [file viruses-12-00857-s001.zip › supplementary/Fig S1.pdf]

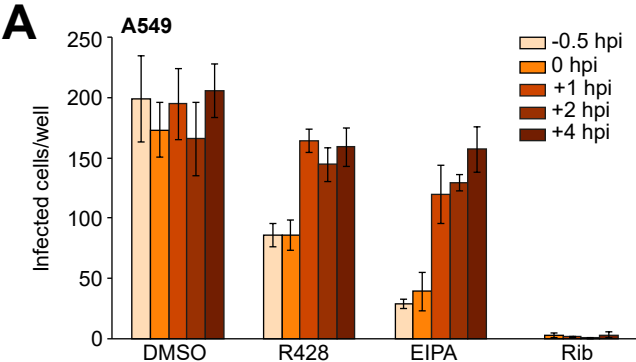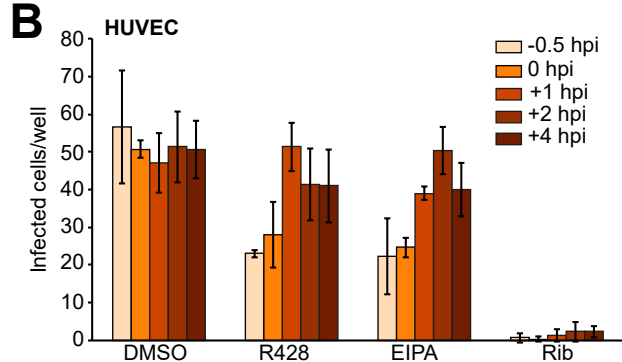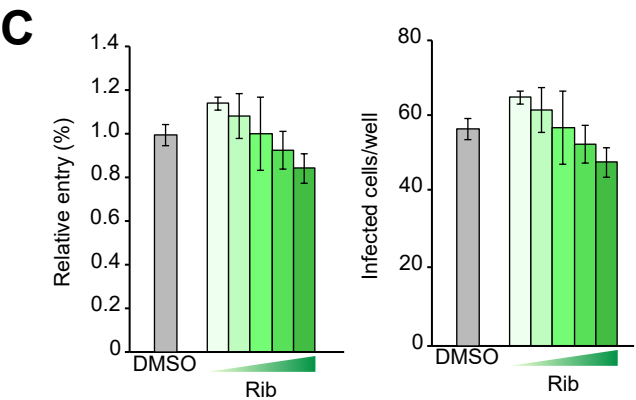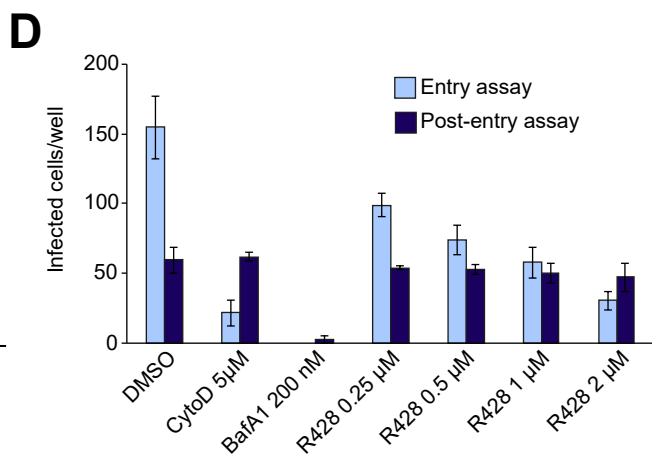

Supplement: Supplementary file 1 [file viruses-12-00857-s001.zip › supplementary/Fig S2.pdf]

**A549**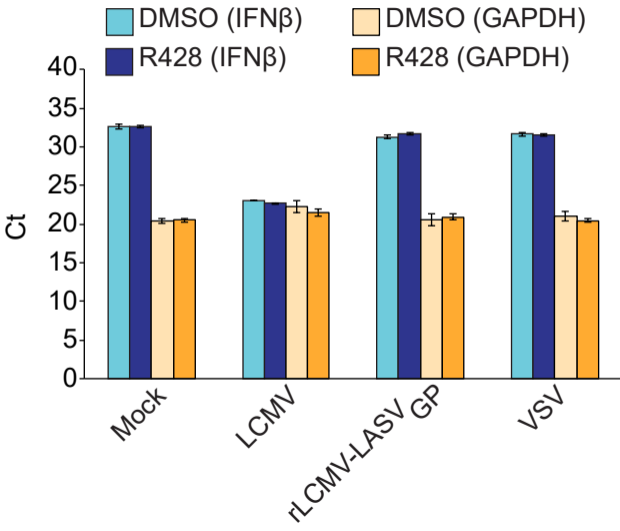**HT-1080**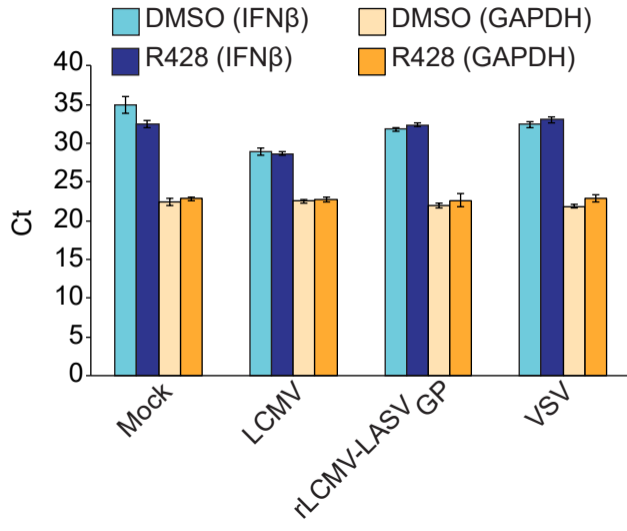

Supplement: Supplementary file 1 [file viruses-12-00857-s001.zip › supplementary/Fig S3.pdf]

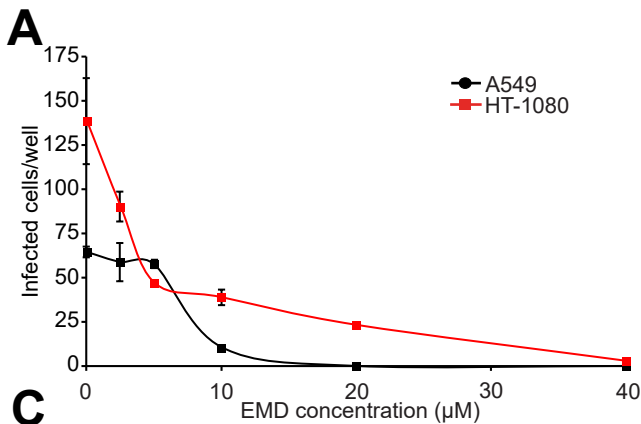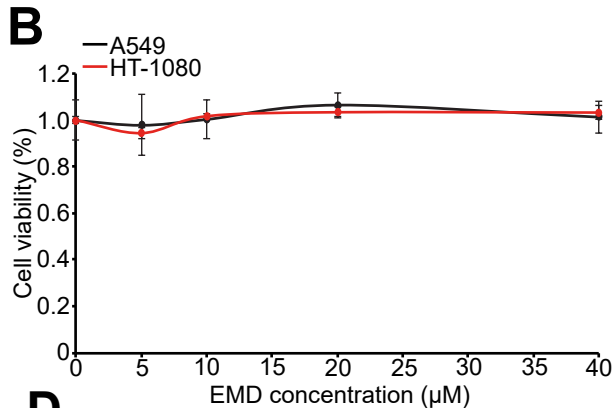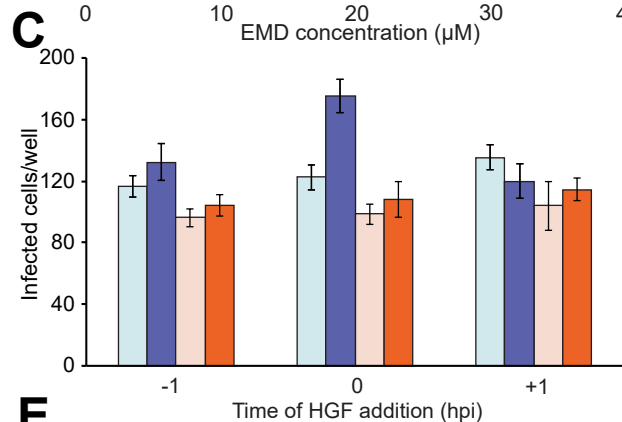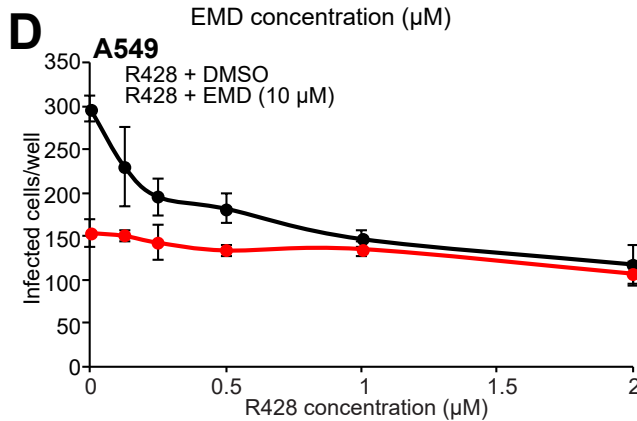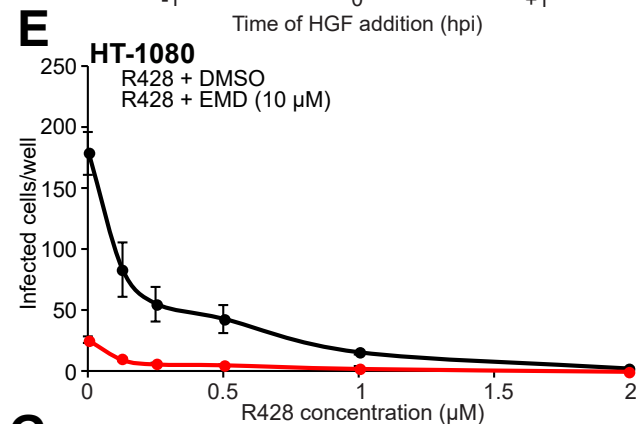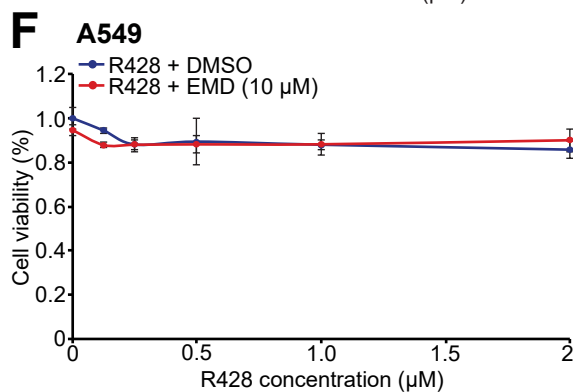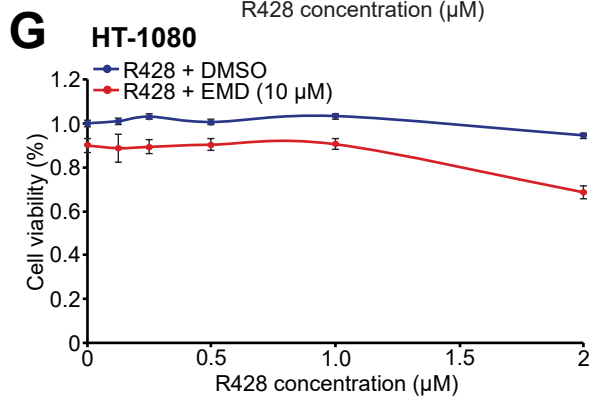

Supplement: Supplementary file 1 [file viruses-12-00857-s001.zip › supplementary/Fig S4.pdf]

Dextran

NP

Merged + DAPI

Uninfected

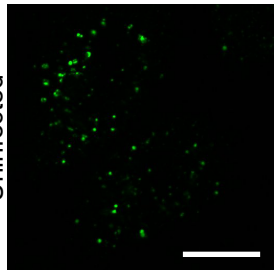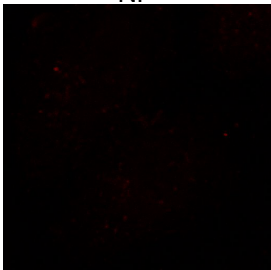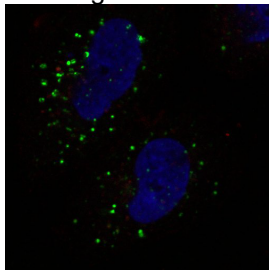

Infected

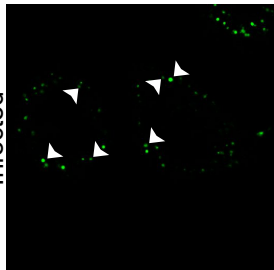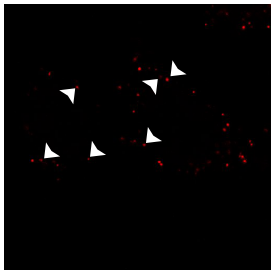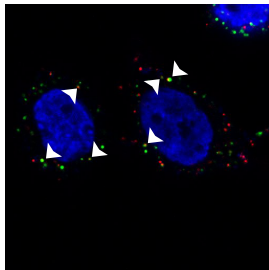

Infected

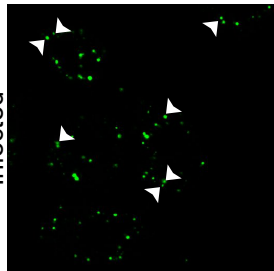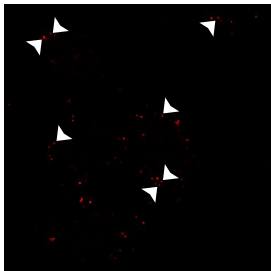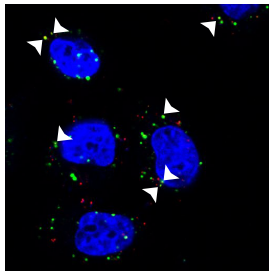

Supplement: Supplementary file 1 [file viruses-12-00857-s001.zip › supplementary/Fig S5.pdf]

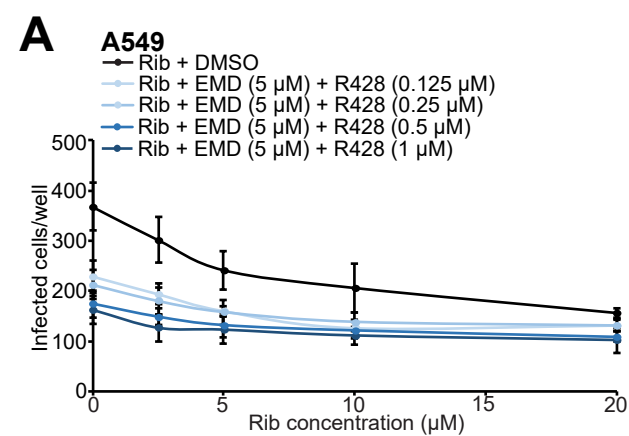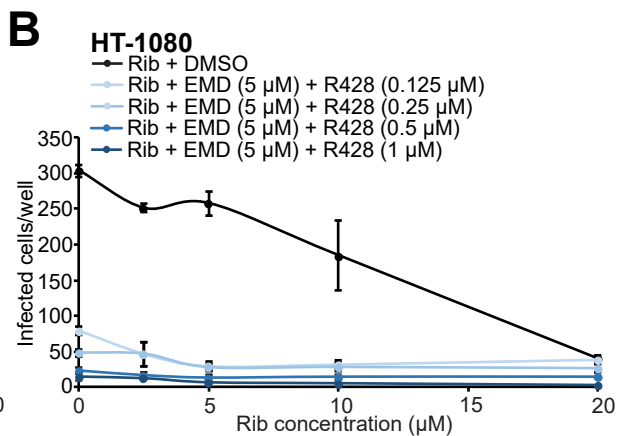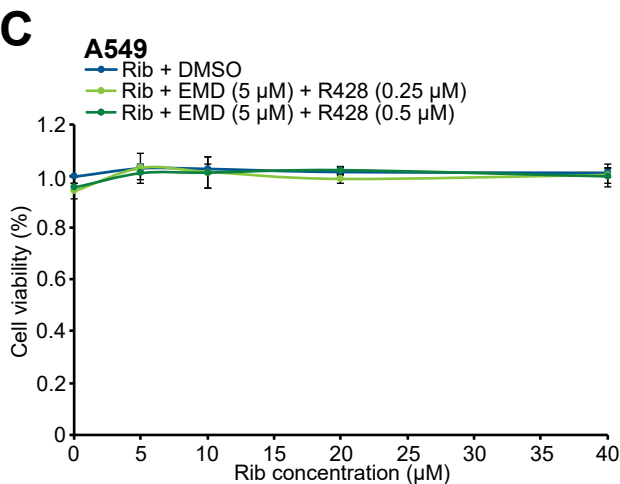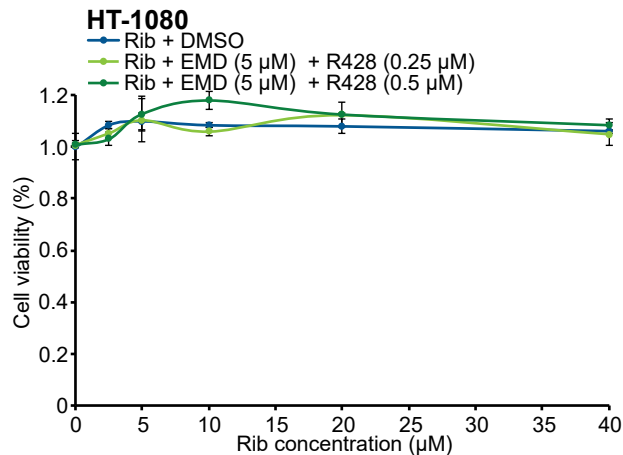

Supplement: Supplementary file 1 [file viruses-12-00857-s001.zip › supplementary/Fig S6.pdf]
